# Effects of cropping patterns on the distribution, carbon contents, and nitrogen contents of aeolian sand soil aggregates in Northwest China

**DOI:** 10.1038/s41598-024-51997-6

**Published:** 2024-01-17

**Authors:** Ziru Niu, Fangjiao An, Yongzhong Su, Juan Li, Tingna Liu

**Affiliations:** 1 Shaanxi Provincial Land Engineering Construction Group, Key Laboratory of Degraded and Unused Land Consolidation Engineering, Ministry of Natural Resources, Xi’an, China; 2 Key Laboratory of Ecological Safety and Sustainable Development in Arid Lands, Northwest Institute of Eco-Environment and Resources, Chinese Academy of Sciences, Beijing, China; 3 Shaanxi Engineering Research Center of Land Consolidation, Shaanxi Provincial Land Consolidation Engineering Technology Research Center, Xi’an, China; 4 School of Civil Engineering, Lanzhou University of Technology, Lanzhou, 730050 China

**Keywords:** Agroecology, Restoration ecology

## Abstract

The long-term physicochemical responses of aeolian sandy soil aggregates to different crop rotation patterns are poorly understood. Here, we collected soil samples from the 0 to 20 cm tillage layer of continuous maize crop and alfalfa–maize rotation plots situated on the edge of the Zhangye Oasis, Northwest China. These samples were analyzed to quantify the influence of both cropping patterns on the structure, carbon content, and nitrogen content of aeolian sandy soils. When compared with long-term continuous maize cropping, planting alfalfa–maize rotation system significantly increased the mass fraction of macro-aggregates with sizes of > 2 mm and 0.25–2 mm from 8.7 to 12.1% and 19.1 to 21.2%, respectively, but decreased the mass fraction of micro-aggregates (0.053–0.25 mm) from 8.1 to 6.2%. Further, there was no significant difference in the content of silt and clay particles between each system. The alfalfa–maize rotation increased the stability of aggregates from 32 to 37%, representing an increase of 15.6%. Soil organic carbon, inorganic carbon, and total nitrogen were mainly enriched in macro-aggregates with sizes of > 2 mm, and silt and clay fractions for both cropping patterns. Implementation of a rotation pattern increased organic carbon contents by 27.2%, 25.6%, 26.7%, and 27.6%, inorganic carbon contents by 14.4%, 4.5%, 53.3%, and 21.0%, and total nitrogen contents by 29.7%, 7.0%, 4.2%, and 50.0% in aggregate particle sizes of > 2 mm, 0.25–2 mm, 0.053–0.25 mm, and < 0.053 mm, respectively, when compared to continuous maize cropping. The alfalfa–maize crop rotation can therefore effectively improve soil aggregate composition and aggregate stability, alongside organic carbon content, inorganic carbon content, total nitrogen content, and their storage capacity. This system thus represents a soil cultivation technique that can increase the soil carbon sequestration capacity in the oasis zone of Northwest China.

## Introduction

As agricultural activities in Northwest China have intensified in recent years, established soil cultivation methods that involve the high-intensity utilization of arable land have caused frequent soil quality problems, including damage to soil structure and a decline in fertility^1–3^. In order to restore and improve soil strength, and to achieve the strategic goal of “storing grain in the land”, the Ministry of Agriculture of China established the “Pilot Program for Exploring the Implementation of Crop Rotation and Fallowing System” in 2016 in order to encourage local farmers to adopt a crop rotation system for cultivation^4^. Crop rotation allows cultivation of a certain plot of land by planting a variety of crops in different cropping cycles. It is also an ecological way to encourage the land to use its own “endogenous technology” to increase soil fertility^5–7^. By varying the types of crops planted in specific locations over time, crop rotation exploits the different ecological demands that different crops require to thrive^8,9^. For example, various crop types flourish in different environments, and require varying amounts of water and nutrients; as such, their rotation improves soil structure, balances the use of soil nutrients, and improves soil fertility, which ultimately brings numerous ecological and economic benefits^10^.

Soil aggregates are the basic units of soil structure, forming due to the combined action of soil microorganisms, plant roots, and complex physicochemical processes^11–14^. The number of aggregates present in a soil is often used to characterize the soil structure, and the abundances and distributions of aggregates are mainly influenced by a soil’s organic carbon content, as well as the cropping system and crop rotation pattern^15,16^. A sound soil structure can enhance soil’s porosity, maintain fertility, and reduce damage caused by wind erosion in arid areas^17^. Soil organic carbon plays a vital role in soil nutrient transformation and is central to changes in a soil’s environmental quality^18–21^. Previous studies have shown that nearly 90% of soil organic carbon in the topsoil of a loose maturing layer exists within the aggregates^22^. Soil aggregates and organic carbon readily interact with each other, as soil aggregates physically protect organic carbon from degradation, and organic carbon promotes the formation and stabilization of aggregates^23^. However, inorganic carbon and organic carbon in soils of the arid zone of Northwest China are coupled to each other, and both participate in the pedological processes that form aggregates^24^. Organic carbon and inorganic carbon are physically protected from microbial decomposition by adsorption onto the surfaces of clay minerals. This adsorption preferentially occurs within macro-aggregates with sizes > 0.25 mm, thus enriching them in carbon and nitrogen, while micro-aggregates with sizes < 0.25 mm remain depleted and preserve the stability of the soil structure^25–27^. Therefore, exploring the relationship between soil aggregates, carbon cycles, and nitrogen cycles for different particle sizes is necessary to develop effective management and control techniques for stabilizing soil aggregates. Meanwhile, aggregate is a structure formed by the adhesion of soil particles and organic matter, and their stability and size have a significant influence on soil structure and function. By separating aggregates, we can deeply understand the formation mechanism and characteristics of different types of aggregates, and analyze the role of different aggregates in the formation of soil structure, so as to provide references for improving soil fertility and organic matter storage, and provide scientific basis for optimizing soil management.

A well-designed cropping pattern can significantly improve soil structure and enhance soil fertility^28^. Compared with rotational cropping patterns, continuous cropping increases the disturbance of soil organic matter in such a way that reduces the occurrence and combination of soil aggregates, thus destabilizing them^29^. In a study of rice fields located in Jiangxi Ganbei, Wang^30^ reported that the proportion of water-stable macro-aggregates significantly increased following replanting and rotation of rice and other crops when compared to continuous cropping of rice. Fan^31^ also compared different cropping patterns in Alluvium in Shanxi, and found that crop rotation significantly enhanced the proportion of aggregates larger than 3 mm and increased their contribution to total organic carbon content. Following a long-term localization experiment of different alfalfa cropping systems in Longzhong, Song^32^ found that a rotational cropping pattern significantly reduced the bulk density and increased the porosity of Huangmian soil, and significantly improved the physical quality of the soil. These results showed that a good cropping system plays an crucial role in improving soil structure and fertility.

Aeolian sandy soils are widely distributed across northwestern China. Generally characterized by low organic matter content, high sand content, poor soil structure, and common nutrient deficiencies, these soils face the challenge of limited water resources in the dry climate of Northwest China. In this context, the alfalfa-maize rotation model can be a better adapt to this drought condition. Maize is one of the crops that are more tolerant to drought conditions, and alfalfa, as a leguminous crop with strong drought adaptability, can not only fix nitrogen, but also improve soil structure and soil fertility. As such, improving their soil fertility and carbon sequestration capacity has become the top priority for local agricultural development^33,34^. The current research aims to address the research gap or knowledge deficiency regarding the effects of crop rotation and continuous cropping on soil structure and nutrients at the regional scale. While previous investigations have primarily focused on fixed-point experiments at agricultural stations, there has been a lack of research conducted on intensive cropping systems in a broader regional context. This study seeks to fill this gap by examining the impact of cropping patterns on soil aggregates, carbon contents, and nitrogen contents in an oasis region located in the Hexi Corridor, Gansu, China. In this study, we studied the Zhangye Oasis, located in the center of the Hexi Corridor, and investigated the long-term effects of seed production in maize that was planted using a continuous cropping technique versus adjacent plots that used a maize–clover rotation pattern. We compared samples and measurements from each area to investigate how the different production methods affected the stability of soil aggregates, and whether long-term maize continuous cropping caused degradation of local soil structure and/or soil fertility. The formation of soil aggregates, soil organic carbon content and nitrogen cycling were emphasized. Using these results, we aimed to (1) investigate the effects of long-term continuous maize cropping and maize–alfalfa rotation on the basic physicochemical properties of aeolian sandy soils, and (2) to analyze how carbon and nitrogen content and storage patterns in soil aggregates change according to cropping pattern. Through this research, we hope to provide scientific basis and technical support for farmland management practice, help improve the planting structure and planting pattern of farmland, and optimize the agricultural production mode in arid areas.

## Materials and methods

### Overview of the study area

**Figure 1:**
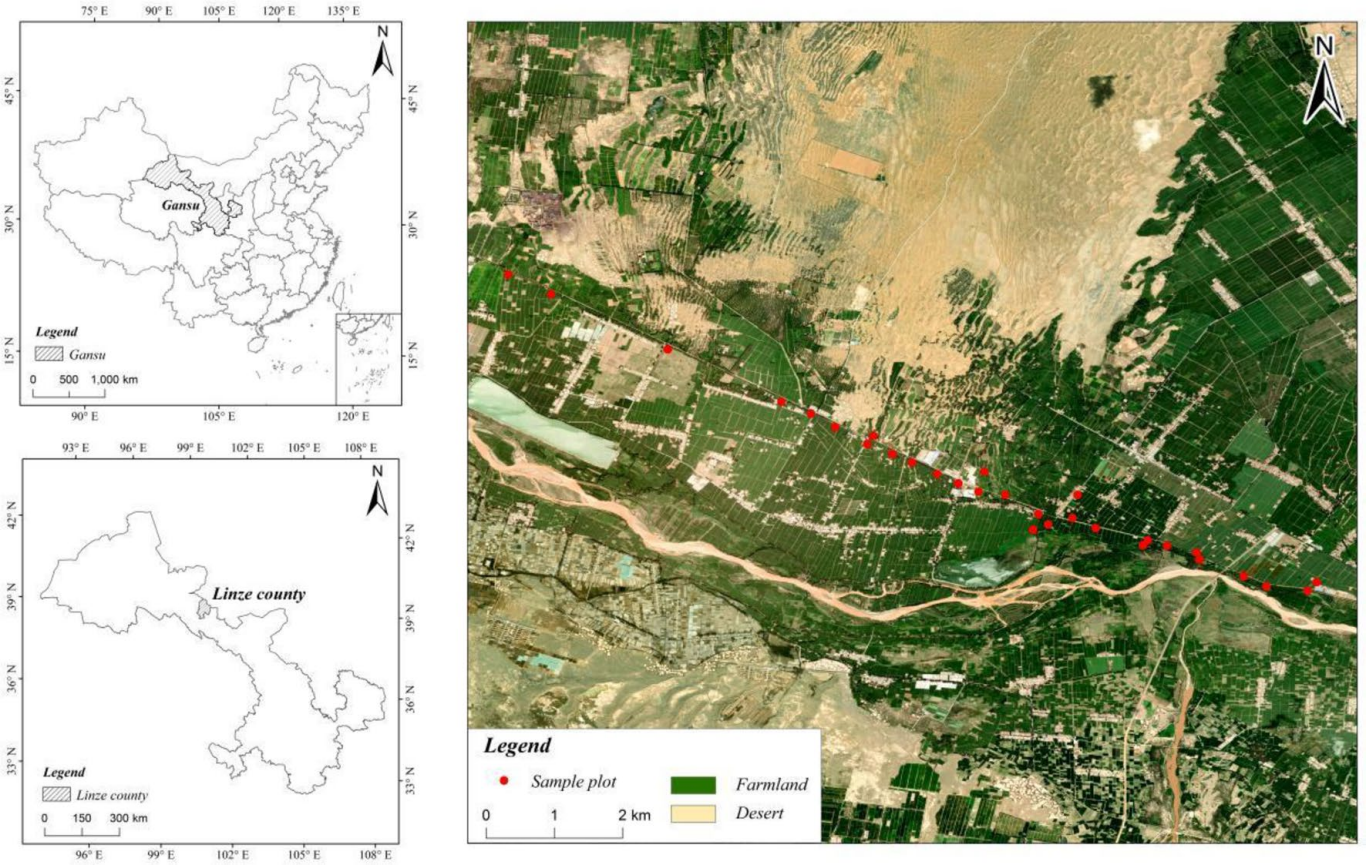
Sampling locations(ArcGIS 10.2, https://www.esri.com/en-us/arcgis/products/arcgis-desktop/resources; Tuxingis, http://www.tuxingis.com/locaspace.html).

All experiments were conducted in an oasis situated on the northern edge of Linze, centrally located within the Hexi Corridor in Gansu (Fig. 1). This region was formerly a desert but has been recently reclaimed and transformed into an oasis. Over the past 30 years, the cultivated land area within the oasis has expanded from 3636 to 4798 km^2^. Surrounded by deserts, including the Gobi Desert, the oasis experiences a typical desert climate based on the Köppen climatic classification system. The climate is characterized by cold winters and dry, hot summers, with an average annual precipitation of 116.8 mm, an annual evaporation of 2390 mm, an average annual temperature of 7.6 °C, and an annual frost-free period lasting 165 days.

In the northern part of the oasis edge, aeolian sand has accumulated over time due to long-term sand invasion and deposition. The soil in this area is classified as an AridiSandic Primosol according to the Chinese Soil Taxonomy classification systems , equivalent to the Aridic Ustipsamments soil in the USDA Soil Taxonomy classification systems. Since desertification control measures were implemented in the late 1960s, the sandy land has been leveled and gradually reclaimed, resulting in sandy irrigated farmland with varying reclamation time series. The irrigation water for farmland mainly relies on the water from Heihe River and groundwater. In recent years, the main crops grown in this area are seed maize and field maize, with traditional tillage and plastic film mulching practices employed. The planting period typically spans from March to September. The number of irrigation cycles during the maize growth period varies from 6 to 11 times due to differences in soil texture. Maize production was 6000 kg ha^−1^, and alfalfa production is 1200 kg ha^−1^. In arid regions, the sandy soil has low fertility and struggles to retain water and fertilizer. As a result, oasis irrigation in this area requires relatively high levels of water consumption.

### Experimental design

In mid-October 2021, we selected 30 long-term continuous crop plots of maize (seeded maize) and 30 adjacent plots planted with alfalfa–maize mixtures in the Pingchuan irrigation area of Linze to form paired samples for subsequent comparison(Fig. 1). These plots were chosen in order to analyze the effects of long-term continuous cropping of maize and the implementation of alfalfa–maize rotation on the stability of soil aggregates, and carbon and nitrogen accumulation. In each plot, soil samples (0–20 cm) were collected from the tillage layer at multiple points within a 50 m^2^ area using a shovel. These samples were mixed and put into bags, which were then boxed up and transported to be analyzed in a laboratory. A sample size of 30 is generally considered a common criterion for meeting the minimum sample size of a statistical normal distribution. More than 30 samples is considered to be a relatively reliable threshold when assuming an approximate normal distribution of data. Finally, 60 representative plots of different cultivation types were selected as sampling sites and further classified by the type of land use: 30 plots had been cultivated continuously for more than 20 years for maize production and 30 plots had been converted to an alfalfa–maize rotation system after 2000–2001.

### Aggregate separation

**Figure 2:**
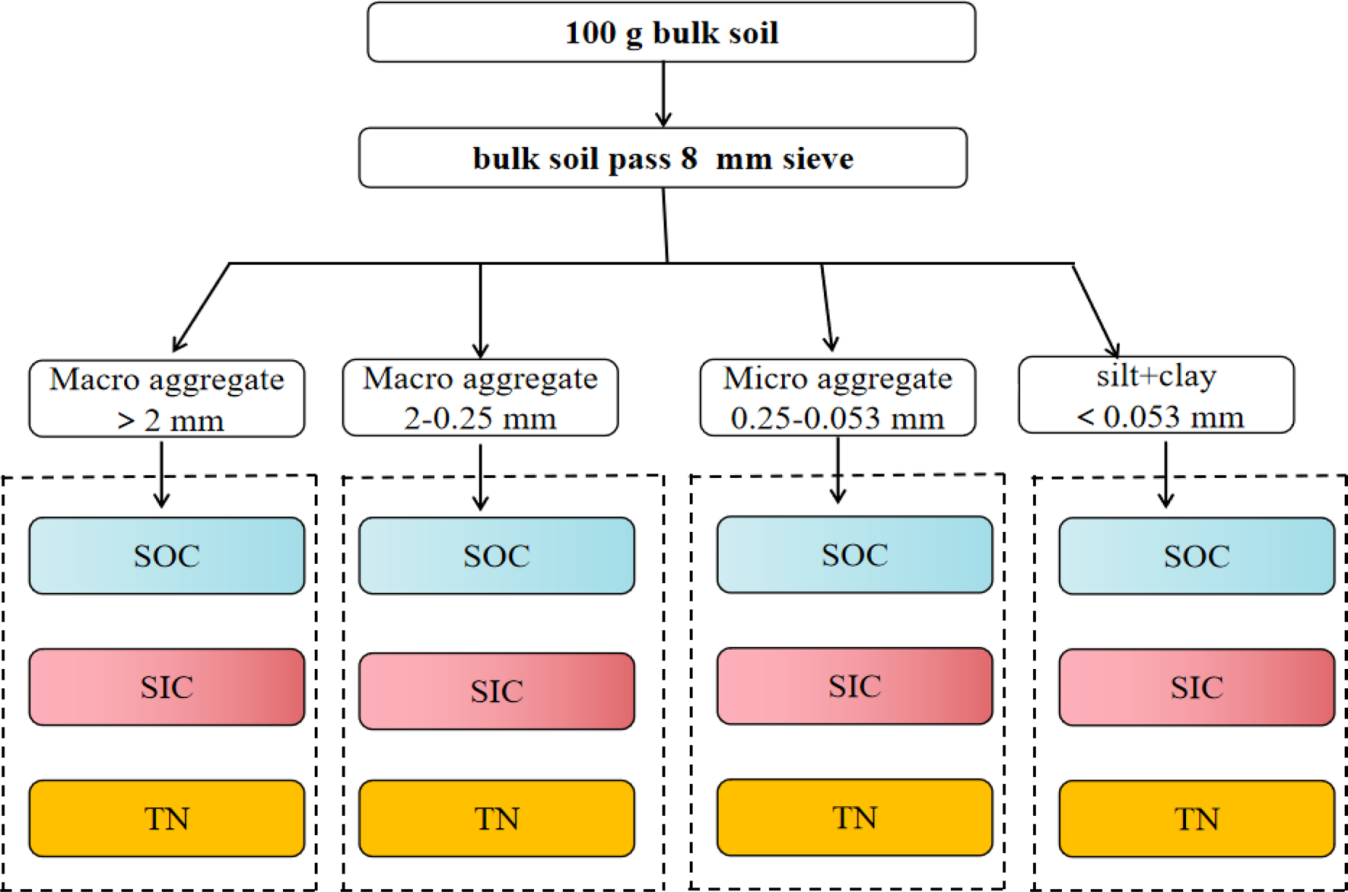
Aggregate fractionation diagram.

Freshly collected soil samples from the field were gently crumbled by hand along natural breaking points and passed through an 8-mm sieve (Fig. 2). Plant and organic debris were carefully removed from the sieved soil with forceps. Once well mixed, a portion was used to study the physical fractionation of the soil, and the remaining portion was air dried and used to determine variations of composition, organic carbon, inorganic carbon, and total nitrogen content according to soil particle size. The soil samples were physically separated into water-stable aggregates of different particle size by the wet sieve method according to Elliott^35^. The test was performed as follows: 100 g of each soil sample was placed in the topmost layer of nested 2-mm, 0.25-mm, and 0.053-mm diameter sieves, pre-saturated in distilled water for 3 min, and then shaken vertically in water 40 times at a rate of 15 oscillations per minute and with an amplitude of 3 cm. The aggregates were then collected from each layer of the nested sieve, transferred to a beaker, dried at 60 °C, and weighed. Since sandy soil aggregates contain a large amount of sand particles, the mass fraction of the aggregates was collected using the method of Six^36^. The previously weighed aggregates were transferred to a beaker and sodium hexametaphosphate was added to separate the aggregates from the sand particles. The sand particles were then removed, the remaining aggregates were transferred to a beaker and dried, and then weighed to obtain accurate mass fractions.

### Determination of the physical and chemical properties of soils

The compositions of soil aggregates with varying particle sizes were determined by the wet sieve and pipette method^37^. Soil total carbon, and the total carbon and total nitrogen content of different particle size aggregates were determined using an Elementar Vario Macro Cube (Germany), and soil organic carbon (SOC) was determined following the Walkley–Black oxidation method using potassium dichromate^38^. Soil pH and EC were determined using a pH meter (BPP-7800). Soil inorganic carbon (SIC) content was obtained by subtracting organic carbon from the measured total carbon for each fraction.

### Data analysis

The mean weight diameter of soil aggregates (MWD) was calculated as follows:

1 $$ MWD = \sum {X{\text{i}} \cdot Wj} $$

where MWD is the mean weight diameter of aggregates (mm), *Xi* is the average diameter of aggregates (mm) in any class range, and *Wj* is the percentage of aggregates corresponding to *Xi*.

Soil aggregate carbon and nitrogen storage capacities were calculated as follows:

2 $$  SOCS = \sum {(Mi \times {\text{SOC}}i) \times BD \times H \times 10}   $$

where *Mi* is the proportion (%) of the i-th size aggregates compared to the total soil volume; *SOCi* is the SOC content of the i-th size (g kg^−1^) aggregates; *BD* is the soil bulk weight (g cm^−3^), and *H* is the soil thickness (cm). Microsoft Excel 2010 and origin18 were used for data processing, performing statistical analysis, and making plots. Data measurements were expressed as mean ± standard deviation. The significance of differences between treatments was analyzed by a one-way ANOVA (α = multiple mean difference test (LSD = 0.05)) using the SPSS software. Pearson correlation analysis was used to analyze the degree of correlation between results using normality test data. The single factor analysis of variance and Pearson correlation analysis were carried out under two planting patterns: maize continuous cropping and alfalfa–maize rotation.

### Ethical approval

The use of plants in the present study complies with international, national and/or institutional guidelines. For the collection of soil and vegetation samples in the study area, we have obtained local permission. The voucher specimen has been placed in the Herbarium of Linze Station to facilitate access to the stored material. Corresponding author Yongzhong Su was responsible for the formal identification of the plant material used in our study. A voucher specimen of the material is stored in the Public Herbarium (ID: 20210521-20210703).

## Results

### Effect of crop rotation and crop succession on the physicochemical properties of aeolian sandy soils

**Figure 3:**
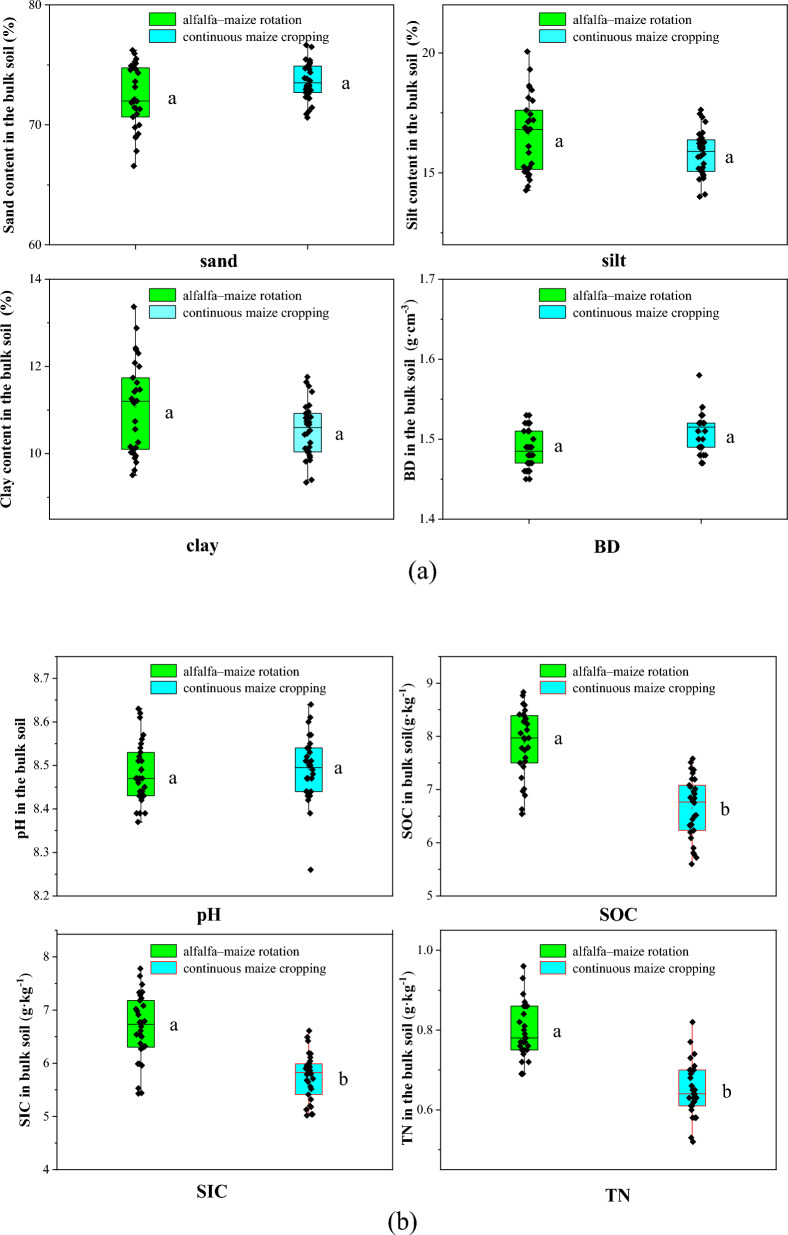
Effects of different planting patterns on soil physical and chemical properties (**a**) physical properties of aeolian-sand soil. (**b**) chemical properties of aeolian sand soil.

The results indicate that crop rotation and continuous cropping patterns did not significantly impact the basic physical properties of the studied aeolian sandy soils (Fig. 3a). Analyses of particle compositions showed that there was no significant difference (*P* > 0.05) in the sand contents of cultivated soil layers (the alfalfa–maize rotation is 72.3% and the continuous cropping is 73.7%, respectively), and no significant difference (*P* > 0.05) in the combined silt and clay contents (27.7% and 26.3%, respectively). After 20 consecutive years of crop rotation, the bulk density of the cultivated layer (0–20 cm) from farmland decreased from 1.54 g cm^−3^ (continuous maize cropping) to 1.48 g cm^−3^ (alfalfa–maize rotation). The bulk density of aeolian sandy soil also decreased by 4.4% during this 20-year period.

Crop rotation and continuous cropping patterns both changed the basic chemical properties of some of the aeolian sandy soils (Fig. 3b). After 15 years of crop rotation, the organic carbon content of soil in the cultivated layer (0–20 cm) under the alfalfa–maize rotation pattern increased significantly from 6.7 g kg^−1^ (continuous maize crop) to 7.9 g kg^−1^ (alfalfa–maize rotation). The organic carbon content also increased by 18.8% during this period. Inorganic carbon and total nitrogen content also increased from 5.8 to 6.7 g kg^−1^ (maize crop) and from 0.7 to 0.8 g kg^−1^ (alfalfa–maize rotation), respectively, which represent increases of 16.0% and 21.5%, respectively. Notably, there was no significant difference in soil pH between the two tillage patterns (*P* > 0.05).

### Effect of crop rotation and continuous cropping patterns on the distribution and stability of aeolian sandy soil aggregates

**Figure 4:**
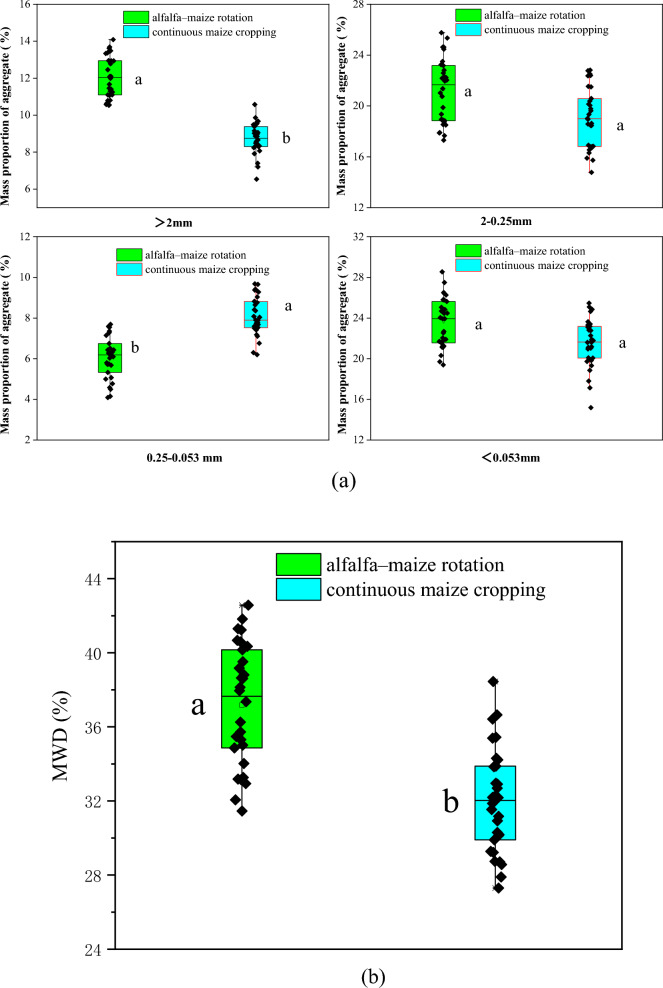
The effect of different planting patterns on the distribution and stability of aggregates (**a**) the effect of planting patterns on the distribution of aggregates (**b**) the effect of planting patterns on the stability of aggregates.

Variations between alfalfa–maize rotation and continuous maize cropping patterns significantly affected soil aggregate formation, distribution, and stability (Fig. 4). When compared with the distribution of sandy soil aggregates in the maize monoculture cropping pattern, the alfalfa–maize rotation significantly increased the mass fraction of > 2 mm macro-aggregates from 8.7 to 12.1%. By contrast, the content of micro-aggregates (0.25–0.053 mm) was significantly reduced from 8.1 to 6.2%(Fig. 4a). There was no significant effect of the planting pattern on the content of combined silt + clay particles. The rotational cropping pattern significantly improved the stability of aeolian sandy soil aggregates from 32 to 37%, representing an increase of 15.6% (Fig. 4b).

### Effect of crop rotation and crop succession on the carbon and nitrogen content of aggregates

**Figure 5:**
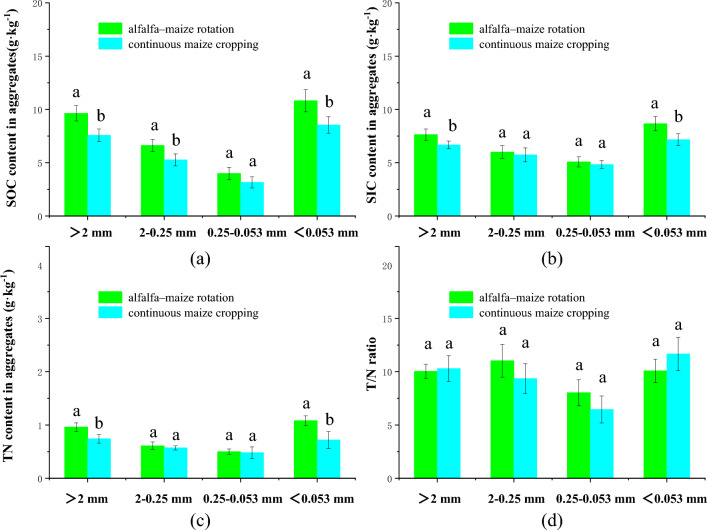
Effects of planting patterns on organic carbon (**a**), inorganic carbon (**b**), total nitrogen (**c**) and T/N ratio (**d**) in aggregates of different sizes.

Crop rotation and continuous cropping patterns significantly affected organic carbon content, inorganic carbon content, total nitrogen content, and the ratio of carbon to nitrogen in soil aggregates (Fig. 5). The mean values of organic carbon content in aggregates for particle sizes > 2 mm, 0.25–2 mm, 0.053–0.25 mm, and < 0.053 mm were 7.6 g kg^−1^, 5.3 g kg^−1^, 3.2 g kg^−1^, and 8.5 g kg^−1^, respectively, in continuous cropped fields, while the mean values of organic carbon content in the same aggregate size fractions were 9.6 g kg^−1^, 6.6 g kg^−1^, and 9.6 g kg^−1^, respectively, in rotational cropped fields(Fig. 5a). The inorganic carbon contents in the aggregates changed from 6.7 g kg^−1^, 5.7 g kg^−1^, 3.2 g kg^−1^, and 7.2 g kg^−1^ to 7.6 g kg^−1^, 6.0 g kg^−1^, 4.8 g kg^−1^, and 8.7 g kg^−1^ in the rotational cropping pattern, respectively, representing increases of 14.4%, 4.5%, 53.3%, and 21.0%(Fig. 5b). In addition, the total nitrogen contents changed from 0.7 g kg^−1^, 0.6 g kg^−1^, 0.5 g kg^−1^, and 0.7 g kg^−1^ to 1.0 g kg^−1^, 0.6 g kg^−1^, 0.5 g kg^−1^, and 1.1 g kg^−1^, respectively, representing increases of 29.7%, 7.0%, 4.2% and 50.0% (Fig. 5c). Despite these changes, there was no significant difference (*P* > 0.05) in the ratio of carbon to nitrogen within aggregates of each particle size between both cropping patterns (Fig. 5d).

### Effect of crop rotation and crop succession on carbon and nitrogen storage in aggregates

**Figure 6:**
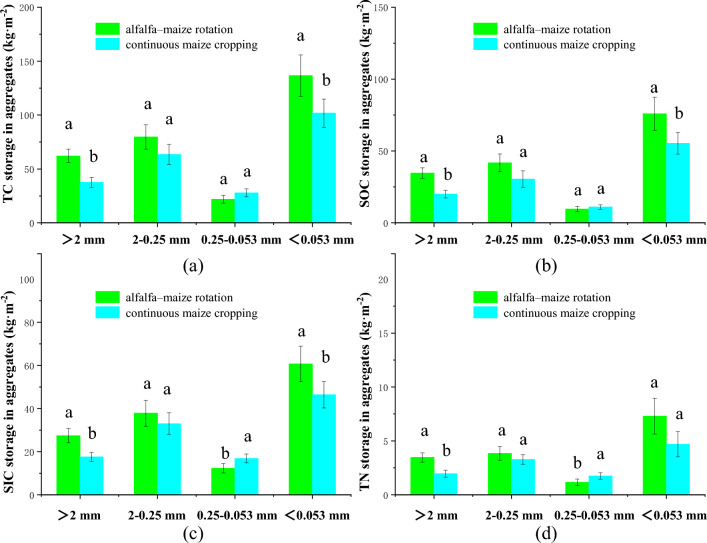
Effects of planting patterns on total carbon (**a**), organic carbon (**b**), inorganic carbon (**c**) and total nitrogen (**d**) storage in aggregates of different sizes.

Crop rotation and continuous cropping patterns significantly affected the total carbon, organic carbon, inorganic carbon, and total nitrogen stocks of the studied aggregates (Fig. 6). The mean values of total carbon stocks in aggregates for particle size ranges of > 2 mm, 0.25–2 mm, 0.053–0.25 mm, and < 0.053 mm were 37.6 kg m^−2^, 63.4 kg m^−2^, 27.8 kg m^−2^, and 101.8 kg m^−2^, respectively, in continuously cropped fields (Fig. 6a). By contrast, total carbon stocks in aggregates for these particle size ranges in rotational cropping patterns were significantly higher, with mean values of 62.0 kg m^−2^, 79.6 kg m^−2^, 21.9 kg m^−2^, and 136.6 kg m^−2^, respectively. For example, total carbon stocks in > 2 mm aggregates, 0.25–2 mm macro-aggregates, and combined silt + clay fractions increased by 65.0%, 25.5%, and 34.3%, respectively; however, total carbon stocks in 0.25-mm micro-aggregates changed from 27.8 to 21.9 kg m^−2^. The organic carbon, inorganic carbon, and total nitrogen stocks in aggregates for each particle size range showed similar trends. The organic carbon stocks in the macro-aggregates and combined silt + clay fractions changed from 20.0 kg m^−2^, 30.4 kg m^−2^, and 55.3 kg m^−2^ to 34.6 kg m^−2^, 41.8 kg m^−2^, 75.9 kg m^−2^, and 55.9 kg m^−2^, respectively, between the rotational cropping pattern and the continuous cropping pattern (Fig. 6b). The organic carbon stock in micro-aggregates changed from 11.0 to 9.6 kg m^−2^ between these two techniques.

The inorganic carbon stocks in macro-aggregates and combined silt + clay particle fractions changed from 17.6 kg m^−2^, 33.0 kg m^−2^, and 46.4 kg m^−2^ to 27.4 kg m^−2^, 37.9 kg m^−2^, and 60.7 kg m^−2^(Fig. 6c). The inorganic carbon stocks in micro-aggregates changed from 16.9 to 12.3 kg m^−2^. The total nitrogen content in macro-aggregates and combined silt + clay particle fractions changed from 2.0 kg m^−2^, 3.3 kg m^−2^, and 4.7 kg m^−2^ to 3.5 kg m^−2^, 3.8 kg m^−2^, and 7.3 kg m^−2^. The total nitrogen storage in micro-aggregates changed from 1.7 to 1.2 kg m^−2^ (Fig. 6d).

## Discussion

### Effect of crop rotation and crop succession on the distribution and stability of aggregates

The composition of soil aggregates significantly affects soil structural stability, soil fertility, and the sustainable production of agroecosystems^27,39,40^. Our research results indicate that compared to continuous maize cropping, adopting a alfalfa-maize rotation helps increase the proportion of macro-aggregates and reduce micro-aggregates in sandy soils, thereby enhancing the stability of soil structure. This finding aligns with Six's study, which observed that an increase in water-stable aggregates significantly improves the stability of soil aggregates and strengthens its erosion resistance^41^. Agricultural management practices have been consistently shown in Bungau’s studies to have a significant impact on the formation and distribution of soil aggregates, with fertilization being one of the key measures^42^. Different fertilization methods, such as green manure crops, no-till straw cover, and the synergistic application of chemical and organic fertilizers, can influence the formation of soil macro-aggregates in various particle size ranges^43^. This suggests that integrated agricultural management practices have a comprehensive impact on soil structure and aggregate formation, playing a crucial role in enhancing soil stability and erosion resistance. For example, continuous planting of green manure crops can increase the formation of macro-aggregates in the 0–20 cm surface layer, no-till straw mulching can increase the content of mechanically stable aggregates in the surface layer and increase the content of water-stable macro-aggregates in the > 0.25 mm size soil, and the combination of chemical fertilizer and organic fertilizer can promote formation of macro-aggregates in soil with particles 0.25–5.00 mm in diameter^44–46^. This is likely because there is a substantial amount of fresh organic residue present in the green manure. These organic materials increase the soluble organic carbon content and microbial activity in the soil, thereby augmenting the binding substances involved in the formation of soil aggregates. Consequently, this process leads to the transformation of some micro-aggregates into macro-aggregates. The results of our study showed that the alfalfa–maize crop rotation pattern significantly increased the mass fraction of macro-aggregates in surface (0–20 cm) soils with particle sizes of > 2 mm and 0.25–2 mm. This indicates that continuous maize cultivation tends to cause fragmentation of macro-aggregates in aeolian sandy soils and converts macro-aggregates to micro-aggregates, which tends to aggravate the reduction of soil pore space. Wei^47^ also showed that a long-cycle crop rotation system in dry farmland on the Loess Plateau (Huangmian soil, Shaanxi, China) significantly increased the number of macro-aggregates in the surface soil with size ranges > 2 mm and 0.25–2 mm. This is consistent with our results that the rotation system increases the number of large aggregates of aeolian soil. Compared to aggregates of different particle sizes, the organic carbon in macro-aggregates is more susceptible to the impact of tillage practices and land use patterns, whereas the organic carbon content in micro-aggregates tends to remain relatively stable. Monoculture planting patterns increase the frequency of soil disturbance through tillage, leading to the continuous mineralization and loss of easily oxidizable organic carbon in the soil. In contrast, the process of crop rotation maintains the original soil structure by reducing soil disturbance, thereby preserving a higher level of stability in soil aggregates. Wang^48^ found that continuous planting of tobacco crops significantly increased the content of < 0.25 mm aggregates in Yunnan’s Alluvium, and significantly reduced the soil’s stability and resistance to erosion over time, which is consistent with our results, the long-term maize continuous cropping system increased the number of micro-aggregates.

Li^49^ showed that the main driving force that reduces aggregate stability and decreases the proportion of water-stable aggregates is a decrease in the organic matter content of a soil. Organic carbon plays an important role in the formation of aggregates, mainly due to its ability to cement particles together. Organic carbon also provides nutrients and energy to soil microorganisms and above-ground crops, allowing them to produce and release more metabolic products into the soil that are used as aggregates for cementation; however, organic carbon itself is also a charged colloid that can adsorb surrounding mineral particles to form aggregates^50^. Following a long-term localization experiment, Li^51^ showed that combining organic fertilizer with nitrogen fertilizer increased the proportion of macro-aggregates in soil, although they found no difference between different fertilization management practices. Gao^52^ reported that the organic carbon content of black kiln soil showed a significant and positive correlation with the proportion of aggregates with particle sizes > 5 mm and 2–5 mm, and showed a strong, significant, and negative correlation with the content of micro-aggregates, which is consistent with the results of this study.

In arid regions, the amounts of organic and inorganic carbon in the soil reflect a balance between the decomposition and synthesis of organic carbon and inorganic carbon^53^. The proportion of inorganic carbon is also closely related to soil aggregate dynamics, as it participates alongside organic carbon in the formation of aggregates^54^. Soil inorganic carbon is mainly present in soil within primary or secondary minerals. Primary carbonates (or rock-forming carbonates) originate from parental rock material^55^. Primary carbonate can be dissolved and transported by water containing organic acids or by reaction with CO_2_ in the soil/atmosphere, and then form secondary carbonate. When a soil’s moisture content decreases or its pH value increases, HCO_3_^−^, dissolved carbonate, and CO_2_ react with free cations to form secondary carbonate^56^. This secondary carbonate wraps around the outside of primary soil particles, allowing it to directly participate in the formation of aggregates.

The MWD is generally considered as an important indicator of soil aggregates’ structural stability, and it is positively correlated with soil aggregation volume, aggregation capacity, and the degree of stability^57^. The results of this study showed that soil aggregation structures in aeolian sandy soils that form via a rotational cropping method were better than those formed during continuous cropping. Indeed, alfalfa–maize rotation significantly increased the MWD of aeolian sandy soils, which agrees with the conclusions of Doetterl^58^. The distribution of crop roots in soil, tillage methods, and soil structure (e.g. pore distribution) are important factors that influence the stability of aggregates. When compared with single-crop continuous cropping techniques, long-cycle crop rotation systems that involve two types of plants increased the variety of soil root secretions, and the replacement of different plant roots facilitated the maintenance of soil microbial diversity. These factors significantly increased the proportion of macro-aggregates and decreased the proportion of micro-aggregates, which improved soil structure and the stability of aggregates by increasing the mass fraction of macro-aggregates with sizes of 0.25–2 mm. Our results showed that crop succession using only maize increased the number of micro-aggregates, which inhibits the formation of macro-aggregates, as also shown by Six ^59^.

Eerd^60^ showed that the soil depth range 0–75 cm is most suitable for root growth, although maize has a root system that extends down to 100 cm. By contrast, the root system of alfalfa generally extends only to a depth of 30 cm. Alfalfa also has slender root cylinders that contain more branches than maize, as it belongs to the legume family, allowing it to fix more nitrogen in the soil. In turn, alfalfa can more efficiently provide microorganisms with energy that they require to produce colloidal material, which is then used to form macro-aggregates^61^. Therefore, long-term crop rotation promotes the formation of macro-aggregates and their stability in the topsoil.

### Effect of crop rotation and crop succession on carbon contents, nitrogen contents, and the storage of aggregates

Soil organic carbon is an important indicator of soil quality and the manner in which it is sequestered in soil largely depends on the physical behavior of aggregates^62^. Organic carbon in macro-aggregates is more susceptible to the effects of tillage systems and land use practices, whereas the organic carbon content within micro-aggregates tends to be more consistent^63^. The nutrients required for plants to grow are drawn mainly from the soil itself, and the removal of large amounts of agricultural products from farmland each year causes a large deficit in effective soil nutrients, causing less organic matter to form via mineralization. Crop rotation changes the amount and type of crop residues or root systems, which affects fixation, mineralization, and the total amount of soil organic carbon that a soil contains. Xue^64^ concluded that the fixation of soil organic carbon is mainly affected by a soil’s microbiota, its structure, and their mutual interactions. The results of this study have shown that an alfalfa–maize rotation pattern increased the content and storage of organic carbon, inorganic carbon, and total nitrogen in all studied topsoil (0–20 cm) macro-aggregates. This may be due to a crop rotation pattern facilitating the continuous change of crops, which increased the biodiversity of the soil’s surface layer^65^. The continuous refreshment of enzymes and microorganisms in a soil promotes organic matter enrichment and the formation of small particles of organic matter inside macro-aggregates, thus increasing the stability of the soil aggregates. Microbial mycelial expansion also helps to form macro-aggregates, such that long-term continuous cropping acts to destabilize structures in soil organic matter and generate more easily decomposable structures. Together, these factors cause organic matter to develop simpler structures over time that are easier to decompose, such that a soil’s carbon content decreases commensurately.

Based on the results of this study, we conclude that adopting a crop rotation system is more advantageous for improving soil fertility compared to a continuous monoculture system. This effect may stem from the increased diversity of soil microorganisms in the crop rotation system, promoting the decomposition of organic matter and the generation of particulate organic carbon and binding substances. This, in turn, facilitates the formation of micro-aggregates into macro-aggregates. The protective coating effect of macro-aggregates helps to preserve organic carbon and total nitrogen, preventing them from being rapidly utilized by microorganisms. As a result, soil nutrients are stored within the macro-aggregates. Zhang^66^ reported that a single and continuous cropping pattern limits the formation of soil aggregates, whereas the adoption of a crop rotation mode is more likely to improve the structure of soil aggregates by increasing soil organic carbon content and stability. This is likely due to the crop rotation mode increasing the diversity of microorganisms in the soil, accelerating the decomposition of organic matter, and generating a significant amount of particulate organic carbon and binding substances, resulting in the formation of macro-aggregates from micro-aggregates. Bai^4^ reported that the wheat–maize rotation promoted the formation of loess aggregates, enhanced their stability, and increased the carbon and nitrogen content of the particles constituting the aggregates. This may be due to the involvement of two plant types in the rotation system, leading to an increased diversity of root exudates in the soil compared to continuous monocropping. Additionally, the alternation of different plant root systems contributes to maintaining soil microbial diversity. All these factors collectively facilitate the accumulation of macro-aggregates. This may be the primary reason for the increase in aeolian sand aggregates in this study due to continuous tillage. Although our data show that macro-aggregates within the 0.25–2 mm size range only had a moderate organic carbon content, they made the largest contribution to soil organic carbon accumulation. This is due to them comprising the largest mass proportion. Additionally, as organic carbon bound by micro-aggregates within the fine macro-aggregate fractions is isolated from microorganisms, it can be preserved and, over time, accumulate significantly, playing a crucial role in the process of soil carbon sequestration^67^.

In this study, we calculated the carbon and nitrogen stocks of aggregates for different particle sizes. Large (> 2 mm) aggregates had the highest whole carbon, organic carbon, inorganic carbon, and whole nitrogen contents, and were dominated by silt + clay, indicating that these two lithological components represent the main sources of soil carbon and nitrogen. This result is consistent with the findings of Henry^68^. The continuous and rotational cropping patterns studied here changed the composition of soil aggregates by altering the organic cementing materials, which in turn influenced the distribution of soil organic carbon in different grain size aggregates. Overall, the alfalfa–maize rotation significantly increased the storage of organic carbon, inorganic carbon, and total nitrogen in macro-aggregates and combined silt + clay fractions in aeolian sandy soils when compared with a single episode of long-term maize cultivation. This result shows that rotation is a more reasonable cropping pattern for carbon and nitrogen sequestration in arid zones.

Our results show that in northwest China, the application of alfalfa maize rotation model can significantly improve the stability of soil aggregates and soil organic matter content compared with continuous maize cropping. The increase in organic carbon and improvement in soil structure can potentially lead to higher agricultural productivity, thereby enhancing crop yields and positively impacting farmers' economic outcomes. At the same time, alfalfa is a high-quality forage, rich in protein, minerals, and vitamins. It can be purchased by local pastoralists, providing a certain economic value. This finding has important implications for soil management practices in the region. However, we also need to be aware that there may be differences between different soil types or regions, as soil properties and climatic conditions can vary significantly in different regions. The study site is only in the desertification area of northwest China, due to the geographical environment and climate conditions in this region and other regions are greatly different, so the extension of the research results in other regions may be limited. Future studies may consider conducting similar experiments in different geographical regions to increase the scope of application of the findings. Comparison of unique cropping patterns, this study focuses on the comparison between the alfalfa–maize rotation model and the single maize continuous cropping model. Future studies can be further expanded to compare the effects of more different crop combinations or crop rotation patterns on soil aggregate structure to identify the most suitable planting patterns in this area.

## Conclusion

Long-term continuous and rotational cropping patterns do not significantly affect the bulk density of aeolian soil, whereas long-term rotational patterns caused organic carbon, inorganic carbon, and total nitrogen content in the soil to increase when compared to long-term continuous cropping. The use of crop rotation or insertion of perennial alfalfa into a continuous crop system can decrease the mass fraction of micro-aggregates, whilst simultaneously increasing the mass fraction and stability of macro-aggregates compared to long-term continuous cropping. Further, the insertion of perennial alfalfa into a continuous cropping system significantly increases the content and storage capacity of organic carbon, inorganic carbon, and total nitrogen in > 0.25 mm macro-aggregates, which makes this a more efficient way to cultivate soil and increase soil carbon sequestration capacity in marginal oasis areas.

## Data Availability

The datasets generated and analysed during the current study are not publicly available due this experiment was a collaborative effort, the trial data does not belong to me alone but are available from the corresponding author on reasonable request.
